# Cut-SOAP: A Machine Learning Descriptor for Rapid Screening of Molecular Adsorption Energetics

**DOI:** 10.1021/acsomega.5c10055

**Published:** 2026-01-26

**Authors:** Felipe V. Calderan, Karla F. Andriani, Priscilla Felício-Sousa, Gabriel A. Pinheiro, Juarez L. F. Da Silva, Marcos G. Quiles

**Affiliations:** † Institute of Science and Technology, 28105 Federal University of São Paulo, Av. Cesare Lattes, 1201, 12247-014 São José dos Campos, SP, Brazil; ‡ Department of Exact Sciences, State University of Santa Cruz, Rod. Ilhéus-Itabuna, 45662-900 Ilhéus, BA, Brazil; § São Carlos Institute of Chemistry, University of São Paulo, Av. Trabalhador São-Carlense 400, 13560-970 São Carlos, SP, Brazil

## Abstract

Adsorption energy is a fundamental property in catalysis and chemical reaction studies; however, conventional quantum chemistry methods, such as density functional theory, provide high accuracy but are often computationally expensive or even impractical for screening large data sets or complex chemical systems. In this work, we proposed a machine learning (ML) pipeline that efficiently predicts relative energy interactions for molecular adsorption near the minimum molecule–cluster distance, at a fraction of the computational cost of quantum chemistry-based methods. Our approach begins by transforming the Fritz–Haber Institute ab initio materials simulation (FHI-aims) output data into feature arrays through a modified version of the Smooth Overlap of Atomic Positions (SOAP) descriptor, which we call Cut-SOAP. This modification reduces the dimensionality of the features by more than 97% while preserving most of the inherent quality of the data. With this method, we construct a large adsorption data set of more than 430,000 entries using real-world data. Then, a deep neural network was trained on this data set, analyzing the influence of architectural and hyperparameter choices on both computational cost and predictive accuracy. The model achieved a mean absolute error below 0.1 eV in the standard test set. To rigorously assess its generalization for real-world applications, we evaluated it on a challenging out-of-distribution data set, where it maintained a robust mean absolute error below 1.0 eV. The trained model is capable of making thousands of predictions in seconds, demonstrating the effectiveness of the pipeline for rapid screening. These results highlight the benefits of ML-based approaches for material screening, which offer accessible, efficient, and accurate tools for predicting relative energy interactions. This capability is a crucial step toward the accelerated discovery and optimization of catalytic systems.

## Graphical abstract

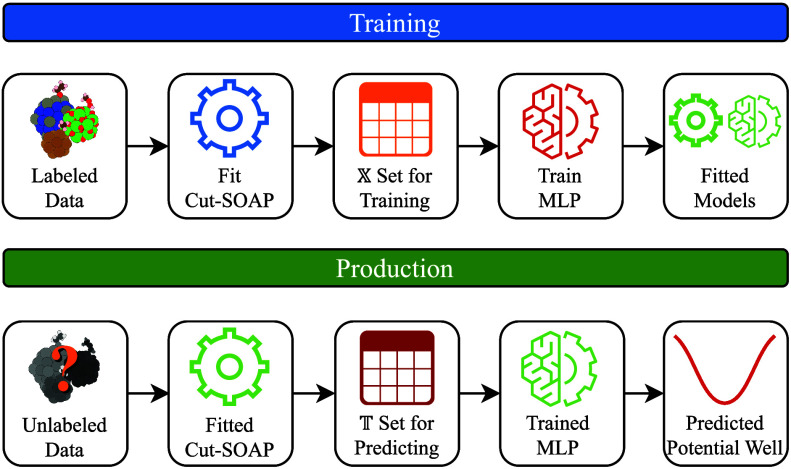

## 1 Introduction

The magnitude of adsorption energy plays a critical role in the domains of surface science, catalysis, and material design because it is a physical chemistry descriptor that quantifies the interaction strength between adsorbates, such as molecules or atoms, and solid surfaces.^1^ Consequently, assessing the magnitude of adsorption energies is of vital importance for a broad spectrum of applications, particularly in heterogeneous catalysis, which includes CH_4_ oxidation,^2,3^ CO_2_ reduction,^4,5^ H_2_ storage,^6,7^ and other essential reactions that take place on surfaces.^8^ Furthermore, the adsorption energy functions as an indirect input parameter in the derivation of linear scaling relationships and the estimation of activation energy barriers via the Bell–Evans–Polanyi (BEP) principle.^9–12^ However, conventional quantum mechanical methodologies, such as density functional theory (DFT), despite their accuracy, require significant computational resources.^13^

Consequently, there exists an increasing demand for methodologies capable of predicting adsorption energies with reduced computational costs, which can help to speed up the estimation of the adsorption energy for a large number of systems at a lower computational cost. Given the continuously expanding corpus of available data, machine learning (ML) techniques constitute a compelling alternative to traditional methodologies, as they exploit readily accessible data to produce predictions with adequate accuracy for numerous applications while operating at considerably enhanced speeds. ML applications in heterogeneous catalysis generally employ bottom-up methodologies, wherein the outcomes of quantum mechanical calculations, specifically DFT, are used to construct designed data sets for ML training. This procedure constitutes the most time-intensive phase within conventional ML workflows, as it requires a considerable number of DFT calculations to obtain an accurate exploration of interaction energies,^14^ which includes the description of all possible molecular orientations on the select surfaces.

In this context, numerous recent investigations have used ML algorithms to forecast the interaction energies of adsorbates on different substrates. Specifically, the works of Toyao et al.^15^ and Zhang and Xu^16^ have used ML models to predict interaction energies for methane-related species in copper-based alloys, taking advantage of the 12 descriptors and properties derived through DFT calculations to train various regression models. Toyao et al. achieved superior accuracy with the application of Extra Forests, attaining a root mean square error (RMSE) below 0.3 eV, while Zhang and Xu documented an RMSE of approximately 0.14 eV using Gaussian process regression.

Furthermore, Chowdhury et al.^17^ applied ML to predict the interaction energies in the context of succinic acid hydrodeoxygenation on six different metal surfaces. They selected fingerprints generated from Simplified Molecular Input Line Entry Specification (SMILES) representations alongside DFT calculations, tabulated values, and counts of chemical bonds as features. Using kernel-based methods, the mean absolute error (MAE) was achieved below 0.16 eV for chain-shaped species and below 0.22 eV for ring-shaped species. Aligned with that, Zong and Vlachos^18^ developed a model using an extreme gradient boost predictor and simple descriptors (valence of the adsorbate, molecular weight, generalized coordination number of the adsorption site, etc.) that do not require DFT to predict the adsorption energy of various species on platinum surfaces. The model demonstrated a good performance across all adsorbates and types of sites, resulting in an RMSE of 0.18 eV.

The increasing integration of ML methodologies within the domain of chemistry has catalyzed the advancement of increasingly sophisticated molecular and material descriptors. These representations are generally classified into two distinct categories: fixed (hand-crafted) and learned (end-to-end) descriptors. Hand-crafted descriptors, such as the eigenvalues of the Coulomb matrix (EVCM),^30,31^ the many-body tensor representation (MBTR),^35^ atom-centered symmetry functions (ACSF),^36^ and the smooth overlap of atomic positions (SOAP),^32^ are explicit mathematical encodings of local atomic environments specifically designed to adhere to fundamental physical symmetries. These representations offer clearly defined interpretability and have historically constituted the foundational framework of ML models aimed at predicting chemical and material properties.

In contrast, end-to-end ML potentials are typically opaque, as they learn their own internal representations from large data sets, without explicit feature engineering. SchNet^19^ is one of the most remarkable works in this regard, as it pioneered the use of continuous-filter convolutions for end-to-end potential learning, which is the cornerstone of many later models. During the same period, Gilmer et al.^20^ formalized the message-passing neural network (MPNN) framework, which became the basis for most subsequent graph-based molecular models. Building upon this paradigm, Schütt et al.^21^ introduced PaiNN, a rotationally equivariant message-passing architecture capable of learning scalar and vector features jointly, enabling the accurate prediction of tensorial properties. More recently, Batatia et al.^23^ introduced MACE, a model that integrates the atomic cluster expansion (ACE) formalism^22^ with equivariant graph neural networks (GNNs).^24^ This hybrid approach achieves high accuracy while requiring a reduced number of layers compared with previous architectures.

Although research in this domain is ongoing, two persistent challenges continue to hinder progress: (i) the lack of intuitive tools accessible to nonprogrammers and (ii) the widespread use of synthetic data sets, which are designed specifically for training ML models.^25–27^ Unfortunately, these issues limit the effective utilization of accumulated knowledge that many research groups already have as well as mask the inherent complexities of real-world applications. These limitations are not without justification. The development of intuitive tools capable of integration with high-performance computing requires substantial software engineering and continuous validation. Moreover, the use of pre-existing databases introduces several complications, such as the need to parse various output formats and address variations in data quality and quantity.

This study presents a comprehensive pipeline for predicting the interaction energies between molecules and clusters near the local minimum configurations that addresses both of the aforementioned challenges. We developed a user-friendly prediction system that seamlessly integrates with command-line environments, that is trained and tested using existing, real-world data sets that were not specifically generated for ML purposes. The current system parses Fritz–Haber Institute ab initio molecular simulation (FHI-aims)^28^ output files (*xyz* and total energies) and then extracts molecular descriptors from the parsed output to organize them into an ML-friendly tabular format. After that, an ML regressor can be selected for training, which includes different options such as linear regression, kernel-ridge regression, and multilayer perceptron (MLP).

Through our computational experimental investigations, we have substantiated the entire pipeline by employing empirical data and have augmented certain computational methodologies previously documented in the literature. Initially, we developed an efficient and precise adaptation of the SOAP descriptor, specifically tailored for the adsorption of molecular species on surfaces, which we have termed Cut-SOAP. Subsequently, we established a simple yet robust neural network that, in tandem with the descriptor, facilitated predictions of high precision. Ultimately, we succeeded in accurately predicting the interaction energies of computer-generated adsorption systems (222) in less than 1 s, illustrating that a comprehensive pipeline based on ML is now feasible within the domain of materials science.

## 2 Theoretical Approach and Computational Details

### 2.1 Quantum Chemistry Database

Our study uses a data set comprising 451,535 molecular configurations (molecules or atoms supported on finite-size particles at different positions and orientations), used primarily for the training and validation of predictive models. The data set encompasses structural details, including *xyz* coordinates, together with the corresponding total energy data derived from DFT calculations. These data were collected from a large number of geometrical optimizations of a wide range of molecular species on several finite-size particles. For example, along geometric optimizations, using a particular local optimizer, a large number of different molecular configurations (*xyz*) are generated, and their respective DFT total energies are calculated. At the end of the optimization, a local minimum configuration is obtained.

All data were obtained through DFT calculations employing the semilocal Perdew–Burke–Ernzerhof (PBE) formulation for the exchange–correlation energy functional. Additionally, the Tkatchenko–Scheffler (TS) correction was applied to enhance the description of long-range van der Waals interactions. The FHI-aims implementation was used along with the light-tier1 and light-tier2 numerical atomic orbitals (FHI-aims terminology). To minimize problems with different levels of accuracy between different basis sets and computational parameters, we employ relative total energies with respect to the molecular species and finite-size systems calculated with the same level of accuracy, respectively.

The data set is characterized by its diversity, which includes a broad spectrum of molecule/substrate-adsorbed configurations. In particular, the collection of adsorbates predominantly consists of molecules commonly encountered in the conversion pathways of methane (CH_4_) and carbon dioxide (CO_2_), such as CH_3_, CO, and CH_2_O, among others. The substrates include a range of finite transition-metal clusters, both unary and binary, with particular emphasis on 3d metals, varying in size from 4 to 55 atoms. Furthermore, the data set comprises doped and undoped oxide clusters, as well as supported clusters and oxides. Figure 2 illustrates the data set’s diversity by showing all molecule–substrate combinations and their corresponding counts.

The study of adsorbed systems reveals the existence of two primary adsorption mechanisms (different magnitudes of the interaction energies): physisorption and chemisorption. These mechanisms are distinguished by the nature of the interactions between the adsorbate molecules and the substrate (finite-size particles). In physisorbed systems, the adsorption energy typically ranges from −0.1 to −0.5 eV, and these systems often exhibit multiple observable adsorption configuration modes. For example, methane (CH_4_) demonstrates three characteristic physisorption modes: umbrella, antiumbrella, and scissoring, with the scissoring mode consistently exhibiting the highest adsorption energy, independent of the substrate’s properties. In contrast, chemisorbed systems are characterized by greater absolute values of adsorption energy and a reduced number of adsorption configuration modes for specific systems. This reduction in modes tends to result in a more uniform energy distribution within the system; e.g., the adsorption of formaldehyde (CH_2_O) on 3d transition-metal clusters (TM_13_) presents fewer adsorption modes, namely, horse-type and Y-shaped configurations, which are associated with the highest and lowest adsorption energy magnitudes, respectively.

These variations reflect the quality of that set, making it a valuable resource for modeling adsorption phenomena through machine learning models. We found that few entries have calculated interaction energies larger than zero, which is undesirable due to physical–chemical implications. These data were removed, leaving the data set with a total of 431,638 valid adsorbed systems.

### 2.2 Data Set Parsing and Formatting

The initial step of this study was the transformation of the outputs of the FHI-aims into an accurately structured table of molecular descriptors. The output files, aims.out, encompass extensive data derived from DFT computations. Consequently, it is crucial to preprocess these output files to extract the requisite information. For this task, we utilized Qcalc, an efficient command-line tool developed in-house to assist in multiple stages of the FHI-aims workflow. Qcalc facilitates the preparation of inputs, including the creation of control.in files and job submission scripts tailored for high-performance computing environments, alongside the postprocessing and analysis of output data. In this study, Qcalc was specifically employed to extract optimized geometries and total energies from the optimization steps documented within the aims.out files.

As mentioned above, the data set contains DFT total energies calculated at different levels of accuracy. In contrast to absolute total energies, relative total energies are significantly less sensitive to computational parameters, such as the basis-set quality, grid density, or numerical convergence thresholds. This robustness makes relative energies more reliable metrics for comparing configurations generated in heterogeneous computational settings. Consequently, we define the relative interaction energy (Δ*E*_tot_) between the molecule (or atom) and the cluster (finite particle size), as depicted in Figure 1a, using

1 $$\Delta E_{\mathrm{tot}}\left(\{R\}\right)=E_{\mathrm{tot}}^{\mathrm{sys}}\left(\{R\}\right)-E_{\mathrm{tot}}^{\mathrm{mol}}\left(\{R\}\right)-E_{\mathrm{tot}}^{\mathrm{cluster}}\left(\{R\}\right)$$

where *E*_tot_^sys^, *E*_tot_^mol^, and *E*_tot_^cluster^ correspond to the total energies of the full adsorption complex (molecule/cluster), the isolated molecule (the lowest energy configuration), and the isolated cluster (the lowest energy configuration), respectively.

**Figure 1:**
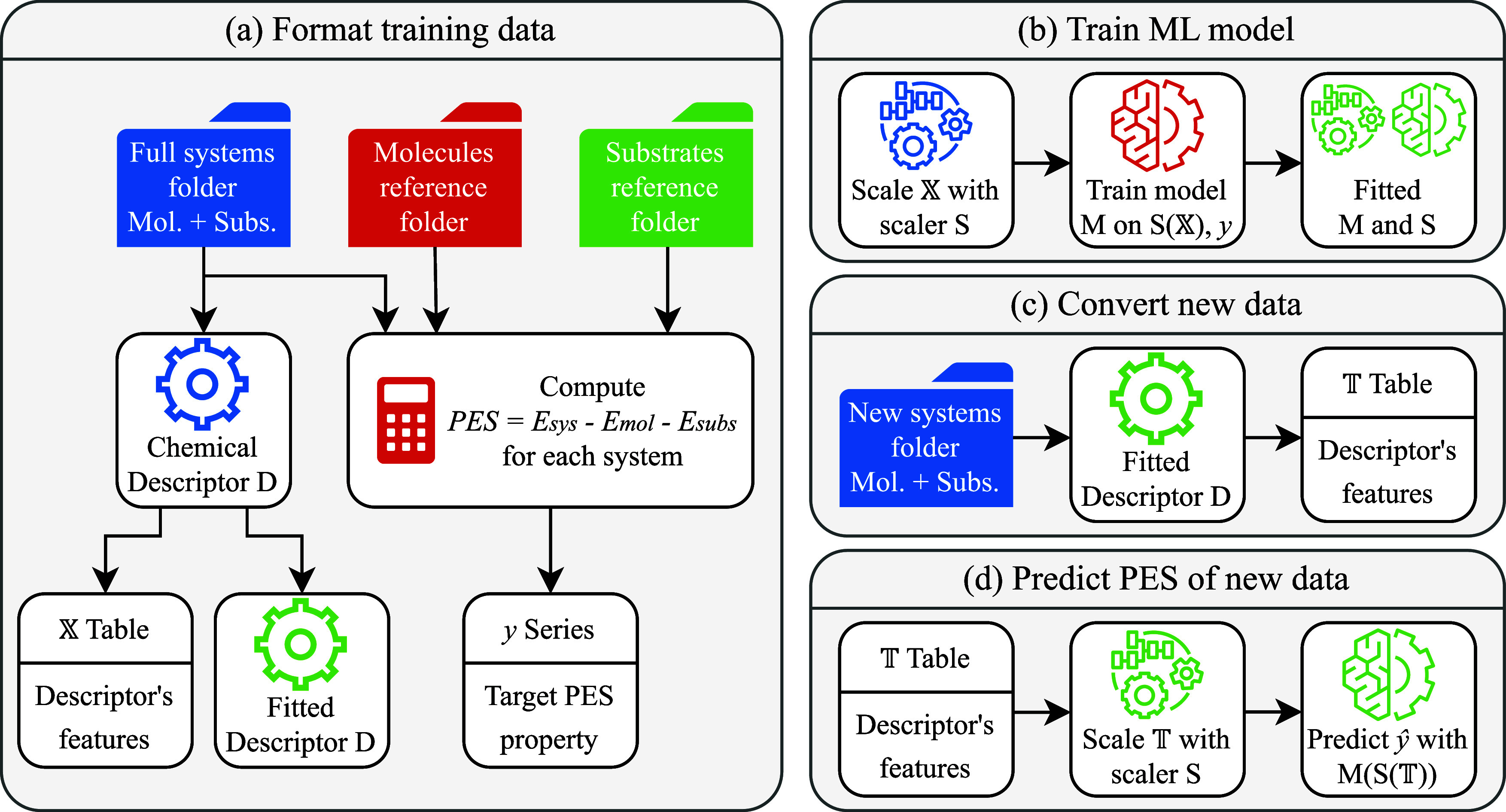
Streamlined representation of the computational pipeline, spanning the initial construction of the data table to the subsequent prediction of new entries. (a–d) Individual software modules. In standard operation, modules (a) and (b) are executed once for the complete data set, whereas modules (c) and (d) are invoked iteratively whenever new data points require prediction.

For each molecule/cluster system under investigation, a comprehensive configurational ensemble is generated through a systematic sampling of diverse molecular orientations, binding motifs, and adsorption sites across the cluster surface. As indicated previously, each system was optimized via local optimization algorithms, resulting in an extensive sampling of the potential energy landscape associated with flexible molecular adsorption. Consequently, the interaction energy Δ*E*_tot_ demonstrates an explicit dependence on the atomic coordinates {**R**}, encapsulating not only the intrinsic strength of the molecule–cluster interaction, but also the geometric variability arising from rotational and conformational degrees of freedom. However, it should be noted that the data set was not specifically designed to map the potential energy surface for ML training purposes.

Because of the nature of the exploration of adsorption sites during geometric optimization, a significant proportion of configurations are situated in proximity to local minimum configurations, resulting in a markedly nonhomogeneous data distribution. This formulation provides a consistent and physically grounded descriptor for the data set, enabling meaningful comparisons between configurations computed at different accuracy levels and serving as a robust target property for machine learning models designed to predict adsorption energetics.

### 2.3 Data Set Rebalancing

Since the data used in this study originate from several distinct projects, it is expected that representation becomes a challenge when training ML models. To address this issue, many of the tests were performed using a rebalanced version of the training set, in which each combination of the molecule and substrate represents at least 1% of the total data set. In other words, rebalancing is a random oversampling of underrepresented classes.

### 2.4 Standard Descriptors

The mathematical procedure that converts chemical information into numerical values within a standardized experimental framework is known as a descriptor, given that the result of this procedure follows some criteria. The numerical values must be invariant with respect to the atom labeling or numbering as well as the rotation and translation of the chemical systems. The procedure also needs to have an unambiguous and algorithmically computable definition and should include a suitable range of values for the molecules described.^29^ Although we analyzed several descriptors, only two were selected for further analysis: the eigenvalues of the Coulomb matrix (EVCM)^30,31^ and SOAP.^32^ These were chosen as the baseline and state-of-the-art methods, respectively. Given our previous knowledge of the systems to which they would be applied, i.e., adsorption systems with diverse characteristics, both descriptors were refined to enhance their effectiveness.

The Coulomb matrix representation is given by eq 2

2 $$C_{\mathrm{ij}}=\{\begin{array}{ll} 0.5Z_{i}^{2.4} & \mathrm{for}\;i=j\\ \frac{Z_{i}Z_{j}}{R_{\mathrm{ij}}} & \mathrm{for}\;i\neq j \end{array}$$

where *Z*_*i*_ and *Z*_*j*_ represent the charges of atoms *i* and *j*, respectively, and *R*_*ij*_ denotes the Euclidean distance between atoms *i* and *j*. To obtain the actual EVCM, which is the true vector of features, we must compute *Cv* = λ*v*, where *v* is the eigenvector and λ is a constant.

EVCM was developed and studied in 2012 and has since become one of the most traditional descriptors derived from molecular geometrical data. Since then, newer and more powerful techniques have emerged. One such example is SOAP. SOAP overlaps atomic positions within a structure using Gaussian functions in a multidimensional space and maps these positions onto coefficients of orthonormal basis functions. This approach enables comparisons of the environments surrounding the central atoms, which are restricted by specified cutoff radii defined as *R*_c_. The partial power spectrum vector (vector of features generated by SOAP) is expressed by eq 3

3 $$p{\left(X\right)}_{b_{1}b_{2}l}^{\alpha \beta }=\pi \sqrt{\frac{8}{2l+1}}\sum_{m}c_{b_{1}\mathrm{lm}}^{\alpha }c_{b_{2}\mathrm{lm}}^{\beta }$$

where X corresponds to the abstract descriptors of atoms around the atom environment from which SOAP was initiated, α and β are environments that highlight particular atomic species, and *l* is the angular degree of spherical harmonics up to a specified maximum value parametrized as *l*_max_. *b*_1_ and *b*_2_ are indices for different radial bases that are limited by a parameter *n*_max_.^33^ The coefficients *c*_nlm_^*Z*^ are defined by eq 4

4 $$c_{\mathrm{nlm}}^{Z}={∭}_{R^{3}}dV\;g_{n}\left(r\right)Y_{\mathrm{lm}}\left(\theta ,\phi \right)p^{Z}\left(r\right)$$

where *g*_*n*_(*r*) is the radial basis function, *Y*_*lm*_(θ, ϕ) are the spherical harmonics, and *p* ^*Z*^(*r*) is the Gaussian smoothed atomic density for atoms with the atomic number *Z*.^34^ It is worth mentioning that the software also supports other standard descriptors, such as MBTR,^35^ local MBTR (LMBTR), and ACSF,^36^ all of which are available through the Python package DScribe.[Fn fna]

### 2.5 Improved Descriptors

The Coulomb matrix presents several challenges because of the variability in the number of atoms across different molecule–substrate systems. Ideally, each entry present in the final table should possess the same number of features, but the number of eigenvalues generated by the EVCM descriptor is the same as the number of atoms. A possibility to address this problem is to zero-fill features in entries with fewer atoms. Unfortunately, this approach introduces a secondary concern since depending on the size difference between elements, unnecessarily huge feature tables can be created, leading to performance degradation. SOAP, on the other hand, presents a different problem that arises from the same fundamental cause. Although it can produce a consistent number of features regardless of atom counts, applying SOAP to systems with large substrates generates an overwhelming number of features, again significantly affecting the computational performance. This phenomenon is due to the rapid increase in the number of atomic interactions as the size of the system grows.
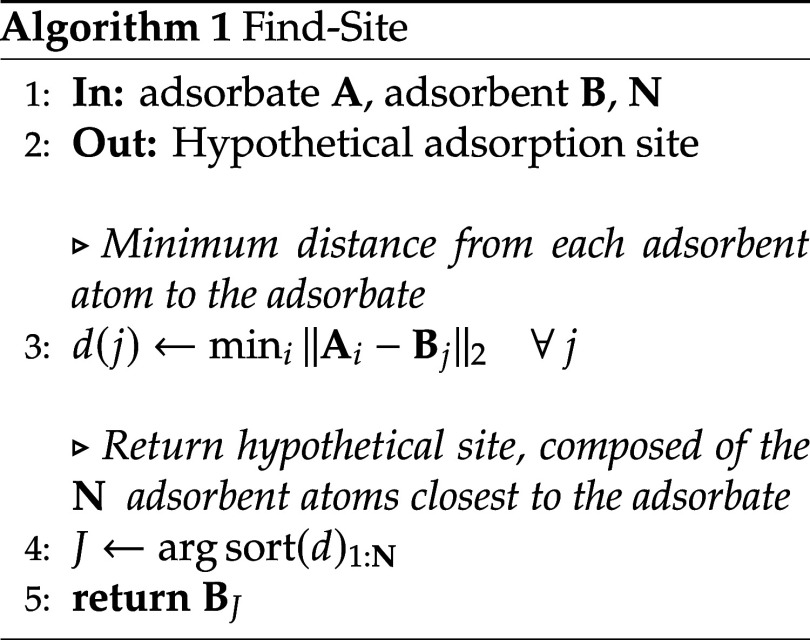


To address these challenges, we developed specialized versions of both descriptors tailored to various adsorption systems. These versions are based on a hypothetical fixed-size adsorption site rather than on whole substrates. The physicochemical implication of this procedure is that the system representation becomes focused on the atomic regions most relevant to the adsorption phenomenon. For EVCM, this standardization equalizes the number of eigenvalues, making zero-filling necessary only for systems with fewer atoms than the hypothetical site. For SOAP, this approach greatly reduces the number of atomic interactions considered, leading to significantly fewer final features.

**Figure 2:**
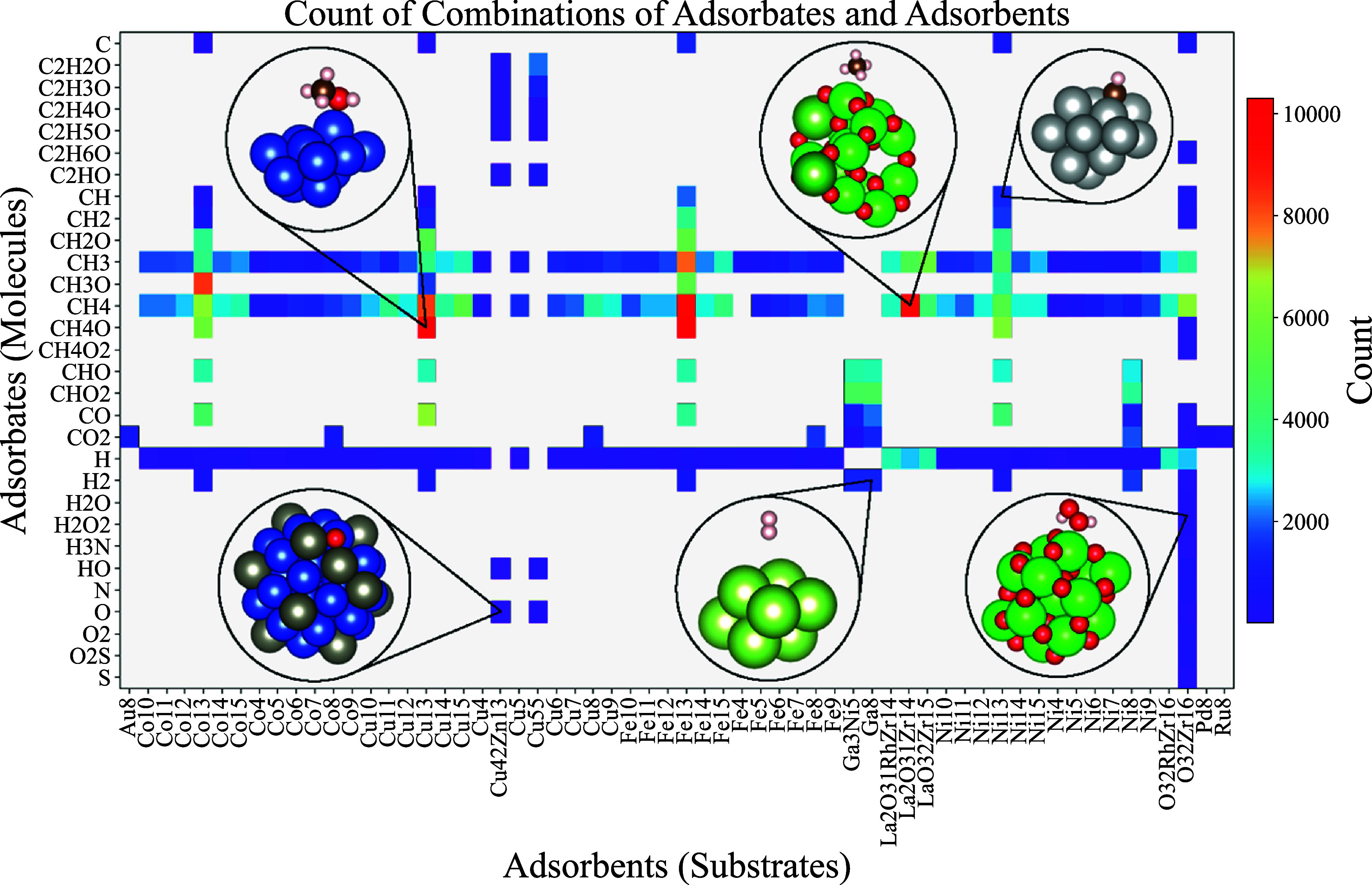
Heatmap representation for the combinations of molecules and substrates comprising the chemical space explored in this study. Several molecular systems are markedly underrepresented relative to others due to the intrinsically organic nature of the data set, resulting in a substantial class imbalance that hinders the training, prediction, and subsequent analysis of the machine learning models.

The hypothetical adsorption sites are constructed by selecting the *N* atoms of the substrate that are closest to the adsorbed molecule, as specified in Algorithm 1 and as illustrated in Figure 3. By applying this procedure, we introduce specialized versions of previously mentioned descriptors: Cut-SOAP and Cut-EVCM. The former is detailed step-by-step in Algorithm 2, while the latter follows the same procedure, differing only in the lack of necessity to compute geometric centers and the EVCM descriptor instead of SOAP. To further reduce the dimensionality of the features, both versions incorporate a correlation analysis to remove highly correlated columns. Table 1 presents the effects of these steps when applied to the default/naïve SOAP descriptor.
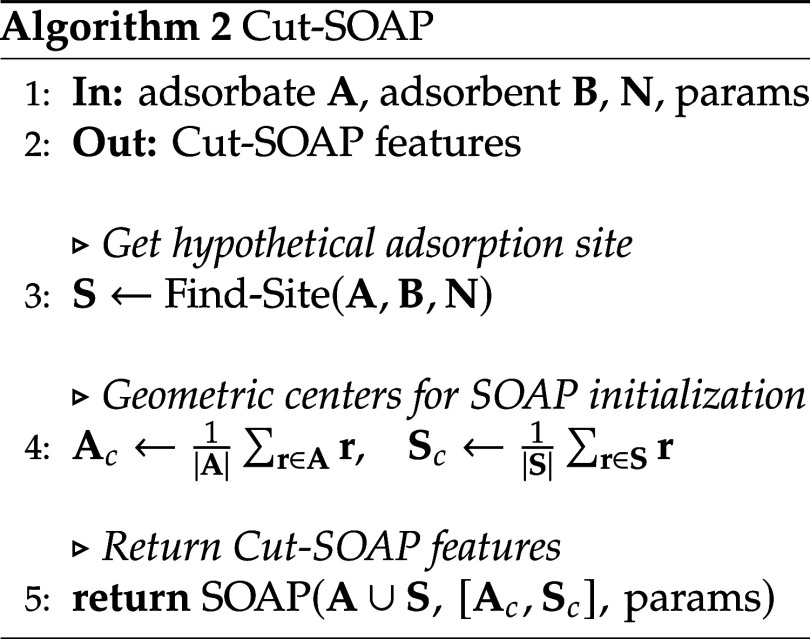


**Figure 3:**
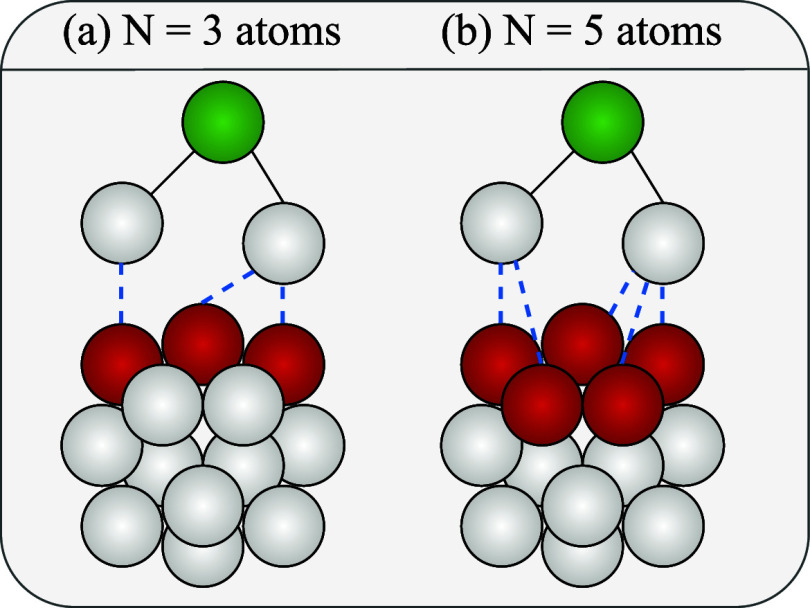
Results of Algorithm 1 for (a) *N* = 3 and (b) *N* = 5 atoms. The atoms highlighted in red form the hypothetical adsorption site, while the green atoms correspond to adsorbate atoms that do not influence the calculation due to their greater distance from the adsorbent.

**Table 1:** Illustration of How Successive Optimizations of the Naïve SOAP Algorithm, Tailoring It for Adsorption Systems, Substantially Reduce the Number of Generated Features^a^

step	resulting algorithm	no. of features
default SOAP with all centers	naïve SOAP	∼75,000
2 centers: molecule and substrate	AA-SOAP	17,850
use the hypothetical site instead of the whole substrate	Cut-SOAP	2380
remove highly correlated features	Cut-SOAP	1835

a The reported values correspond to the total number of features obtained after fitting each SOAP variant to the entire data set. The final version, Cut-SOAP with correlation analysis, achieves a 97.5% reduction in feature dimensionality.

### 2.6 Regressor Models

The most basic regression algorithm is linear regression.^37^ If we consider the chemical data set as a function that maps from the descriptor vector to the relative energy interactions, a linear regression model attempts to fit an *n*-dimensional hyperplane within the feature space to better predict the desired properties. However, the core assumption of a linear relationship is often too simple for the complex, nonlinear nature of large chemical systems, leading to unsatisfactory accuracy. Despite this limitation, its simplicity and computational efficiency make it an indispensable baseline for benchmarking more sophisticated regression algorithms.

The multilayer perceptron (MLP) neural network is a regressor model that employs chained perceptrons^38^ (also commonly referred to as neurons) in a configuration that allows them to be trained^39^ to minimize prediction error. The MLP training process typically involves some kind of backpropagation^40^ through the use of an optimizer. Two widely used optimizers in the literature are Stochastic Gradient Descent (SGD)^41^ and Adam.^42^ These optimizers minimize a given error function, such as the MAE. In essence, after the signal has propagated to the output layer, the error at each neuron is retroactively computed, and fine adjustments to the network’s weights are made accordingly.

## 3 Results and Discussion

In this section, we begin by introducing the evaluation metric used to compare prediction accuracy. Next, we demonstrate that an MLP significantly outperforms the baseline linear regression model for this task. We then compare several chemical descriptors in terms of both computational cost and predictive accuracy, using the previously established MLP architecture. Once the most cost-effective descriptor tested is identified, we further investigate its performance under different parameter configurations. Finally, by combining the better descriptor with the MLP, we fine-tune the hyperparameters of the regressor to minimize prediction errors. The outcome of this process is an accurate and computationally efficient model tailored to our problem.

### 3.1 Computational Environment

The experimental procedures were executed on a computational system equipped with an Intel Xeon Silver 4214R processor operating at a frequency of 2.40 GHz. The system was also configured with 256 GB of DDR4 to 3200 MHz RAM, distributed in eight 32 GB modules. Storage capabilities included a 12 GB s^–1^, 7200 rpm, 8 TB SAS drive. The operating system utilized was Ubuntu 20.04 LTS.

### 3.2 Evaluation Metric

In 2005, Willmott and Matsuura^43^ argued that the MAE offers a more intuitive and unambiguous measure of the average model error compared to the RMSE. In contrast, Chai and Draxler^44^ later contended that the RMSE is more informative when model errors follow an approximately normal distribution, as it better reflects larger deviations due to its quadratic formulation. The MAE is given by eq 5

5 $$\mathrm{MAE}=\frac{1}{n}\sum_{i=1}^{n}\left|y_{i}-{ŷ}_{i}\right|$$

where *n* is the number of elements to compare, *y*_*i*_ is the real value, and *ŷ*_*i*_ is the predicted value. The RMSE is given by eq 6

6 $$\mathrm{RMSE}=\sqrt{\frac{1}{n}\sum_{i=1}^{n}{\left(y_{i}-{ŷ}_{i}\right)}^{2}}$$

Since errors did not follow a normal distribution in our tests, we preferred to use the MAE for model evaluation. Another important point is that the error distribution for Cut-SOAP + MLP is leptokurtic. In our case, this is beneficial since it is better to make many very accurate predictions, even if a few present significant errors, as the overall outcome remains overwhelmingly good.

### 3.3 Data Set Split

In supervised machine learning, three distinct data sets are generally employed: the training set, the validation set, and the test set. The training set is used to optimize the model’s parameters through iterative learning procedures. The validation set serves to monitor the model’s performance during training, providing an independent measure for hyperparameter tuning. In contrast, the test set is completely withheld throughout the training process and used exclusively after the model has been fully trained. This separation ensures that the test set provides an unbiased estimate of the generalizability of the model.

For the experiments conducted in this study, the data set was initially partitioned into a development set and a test set, comprising 95 and 5% of the data, respectively. For models requiring a separate validation set, the development set was further divided into a training set and a validation set in a 90–10% ratio. This procedure resulted in three mutually exclusive subsets: training, validation, and test.

### 3.4 MLP vs Linear Models

Throughout the various tests performed in this project, we employed several MLP models with different hyperparameters to predict the relative energy interactions. However, it is first necessary to assess to what extent the system really benefits from the use of a more complex model. Therefore, it is prudent to first compare the quality of the predictions against a baseline linear regression model. For this comparison, we used the baseline Cut-EVCM descriptor to convert the *xyz* structural data into an ML-friendly tabular format. The descriptor was set to use six substrate atoms to generate the hypothetical adsorption site. The MLP was configured with four hidden layers containing 1024, 256, 64, and 8 neurons, respectively. It used the ReLU activation function and the Adam optimizer with a learning rate of 1 × 10^–3^. The model was trained for 500 epochs using a batch size of 10,000.

The features exhibiting a correlation ratio of 95% or higher with other features were removed. For this basic test, no rebalancing or data augmentation was performed on the data set. The linear regressor model predicted the test data with a MAE of 0.919 eV, while the MLP achieved a significantly lower MAE of 0.157 eV. Although the linear regression machine required only 11 s to train, compared to almost 18 min for the MLP, the substantial improvement in accuracy makes the additional time worthwhile. Furthermore, completing training under 18 for more than 410,000 entries remains remarkably fast.

### 3.5 Performance Comparison between Various Descriptors

Here, we evaluated several SOAP-based methods in conjunction with our baseline Cut-EVCM using the same neural network, as described in Section 3.4. Before presenting the results for each descriptor tested, it is important to note that running a completely naïve SOAP (starting on all atoms of the system) generates over 75,000 features, which is computationally prohibitive. Therefore, feature reduction techniques were essential.

The descriptors tested include Cut-EVCM, SOAP, Cut-SOAP, AA-SOAP (all-atoms SOAP), and SP-SOAP (single-point SOAP). For Cut-SOAP, we tested two variants, Cut-SOAP 0 and Cut-SOAP 2, which have different parameters for the cut radius and *n*_max_. In this context, SOAP represents the naïve SOAP approach but is limited to only 2 starting points: one in the molecule and the other in the substrate. The same 2-starting-point approach was applied to Cut-SOAP, though in this case, the descriptor was restricted to a hypothetical adsorption site. AA-SOAP is similar but uses all atoms in the molecule and the hypothetical site, instead of 1 single representative atom of each part. Finally, SP-SOAP employs a single starting point located at the interface between the molecule and the substrate.

The evaluation presented in Table 2 reveals that the AA-SOAP descriptor is computationally more demanding than the alternatives and the Cut-EVCM descriptor yields lower predictive accuracy. In contrast, Cut-SOAP 0, Cut-SOAP 2, SOAP, and SP-SOAP all demonstrate a favorable balance between computational efficiency and predictive precision. Therefore, the selection of an optimal descriptor is dependent on the specific characteristics of the data set and the desired trade-off between computational cost and model precision.

**Table 2:** For Each Descriptor Compared, the Time Taken to Build the Training Table, the Time to Train the MLP Using the Table and its Rebalanced (rbl) Version, and the Same for the Mean Absolute Error (MAE) of the Relative Energy Interaction Predictions

descriptor	build time (min)	train time (min)	train (rbl) (min)	MAE (eV)	MAE (rbl) (eV)
AA-SOAP^a^	5361.26	202.15		0.065	
C-EVCM	26.54	13.97	32.86	0.194	0.194
C-SOAP 0	17.49	16.91	50.61	0.106	0.103
C-SOAP 2	116.45	51.95	122.85	0.075	0.062
SOAP	111.22	59.15	150.46	0.076	0.064
SP-SOAP	41.91	37.96	101.28	0.090	0.082

a Tables for AA-SOAP using the rebalanced training set were not generated due to the computational cost.

More details on the parameter settings for each descriptor are reported in the Supporting Information, including additional figures and commentary on the relationship between feature dimensionality and computational cost.

### 3.6 Best Cut-SOAP Settings

Based on previous tests, we selected Cut-SOAP 2 as a descriptor for further analysis, as it yielded the best results for the rebalanced data set while maintaining a reasonable computational cost. Although selecting Cut-SOAP 2 implies that many of the optimal settings are already known, it is still necessary to evaluate the impact of varying the number of atoms (*n*) used to define the hypothetical adsorption site.

Using an augmented version of the data set, as explained in Section 2.3, the Cut-SOAP 2 descriptor, and the same MLP structure shown in Section 3.4, we generated Table 3 by varying the site_size (*n*) from 1 to 8. The result shows that the runs with *n* > 3 produce a very predictive performance, with *n* = 6 producing the lowest MAE prediction. In terms of model construction and training time, all configurations are also comparable with only minor fluctuations.

**Table 3:** How Different Site Sizes (*n*) Affect the Build Time, Train Time, and Prediction MAE of Cut-SOAP 2

*n*	build time (min)	train time (min)	pred. MAE
1	119.74	170.84	0.15
2	116.12	164.10	1.24
3	116.58	160.95	0.07
4	114.62	136.71	0.06
5	117.14	162.74	0.06
6	115.19	114.59	0.06
7	115.68	170.12	0.06
8	114.59	170.98	0.06

### 3.7 MLP Hyperparameters

After optimizing Cut-SOAP’s settings to achieve the best results for the given data set, the final step in building a fast and accurate model is to optimize the MLP regressor. Table 4 shows how different hyperparameter configurations affect the training time, the absolute mean of the prediction, and the variance of the prediction. The labels assigned to each MLP type are used solely for the sake of convenience. The Standard network is the same as shown in Section 3.4, while the Flat and Large variations have less and more neurons, respectively. MiniBatch uses very small batch sizes; Patient can tolerate some epochs of increasing the MAE before stopping early; and Combo integrates characteristics from all other variants. Based on the compiled data, several conclusions can be drawn by comparing the different MLP settings to the standard model.

**Table 4:** How Different Topologies and Hyperparameters for the MLP Affect the Train Time and Prediction Statistics

Measurement	Standard	Flat	MiniBatch	Large	Patient	Combo
train time (min)	124.05	12.02	930.68	406.58	513.72	1547.15
pred. MAE	0.059	0.094	0.064	0.049	0.059	0.049
pred. VAR	0.037	0.052	0.037	0.036	0.038	0.036

An analysis of several network architectures revealed critical trade-offs between computational cost and predictive accuracy. The Flat variant, while efficient, produced models with inferior accuracy, whereas the MiniBatch and Patient configurations increased the training time without offering a commensurate improvement in performance. The most promising results were from Large and Combo architectures. Although the Combo model achieved the highest absolute accuracy, its computational cost was substantial. Crucially, this marginal increase in accuracy over that of the Large model did not justify the disproportionately longer training time. Therefore, the Large architecture was selected for all subsequent investigations, as it represents the optimal compromise between predictive power and computational efficiency.

## 4 Assessment of the ML Model on Real-Life Applications

To validate the model in a realistic application context, we generated completely new adsorption systems 222. These systems were constructed to fall within the expected range of distances between the adsorbate and the adsorbent, as learned by the neural network, while allowing for complete freedom in the orientation of the molecule. This setup simulates a practical scenario in which a specialist obtains data entries from a high-throughput screening process and wants to rapidly evaluate their relative energy interactions.

We selected the adsorption of CH_4_, CH_3_, and H species on (ZrO_2_)_16_-based substrates. Accurate DFT calculations and *xyz* data for these systems will be available in the upcoming work. The adsorbed systems were generated by modifying Module 3 (Mod3) of the cluster_assembler framework to simulate the adsorption of a single species.^45^ Each species was randomly distributed around preoptimized (ZrO_2_)_16_-based substrates at expected distances, previously determined through data set analysis, i.e., boxplot-derived distances. For each simulation run, using a fixed predetermined distance, 10,000 configurations were generated and internally clustered using the *k*-means algorithm, employing the setting RUN_MOD_ZERO = true. All other parameters were maintained at their default values. From these simulations, we obtained representative configurations of 222 for the adsorption of CH_4_, CH_3_, and H on the substrates Zr_16_O_32_, RhZr_16_O_32_, LaZr_15_O_32_, RhLa_2_Zr_14_O_31_, and La_2_Zr_14_O_31_.

Using the saved SOAP descriptor from the earlier training steps, we were able to convert, in less than 1 s, all *xyz* files into a table containing 222 systems (rows), each with 1835 features (columns). This is the exact format expected from our trained neural network. Predicting the relative energy for all generated structures took only 0.28 s, and compared to the reference values obtained from the DFT calculations, the model achieved an overall MAE of 0.892 eV. Individual MAEs for each type of system (with negative relative energy interactions) are shown in Table 5, and a comparison between predicted and true relative energy interactions values is illustrated in Figure 4.

**Figure 4:**
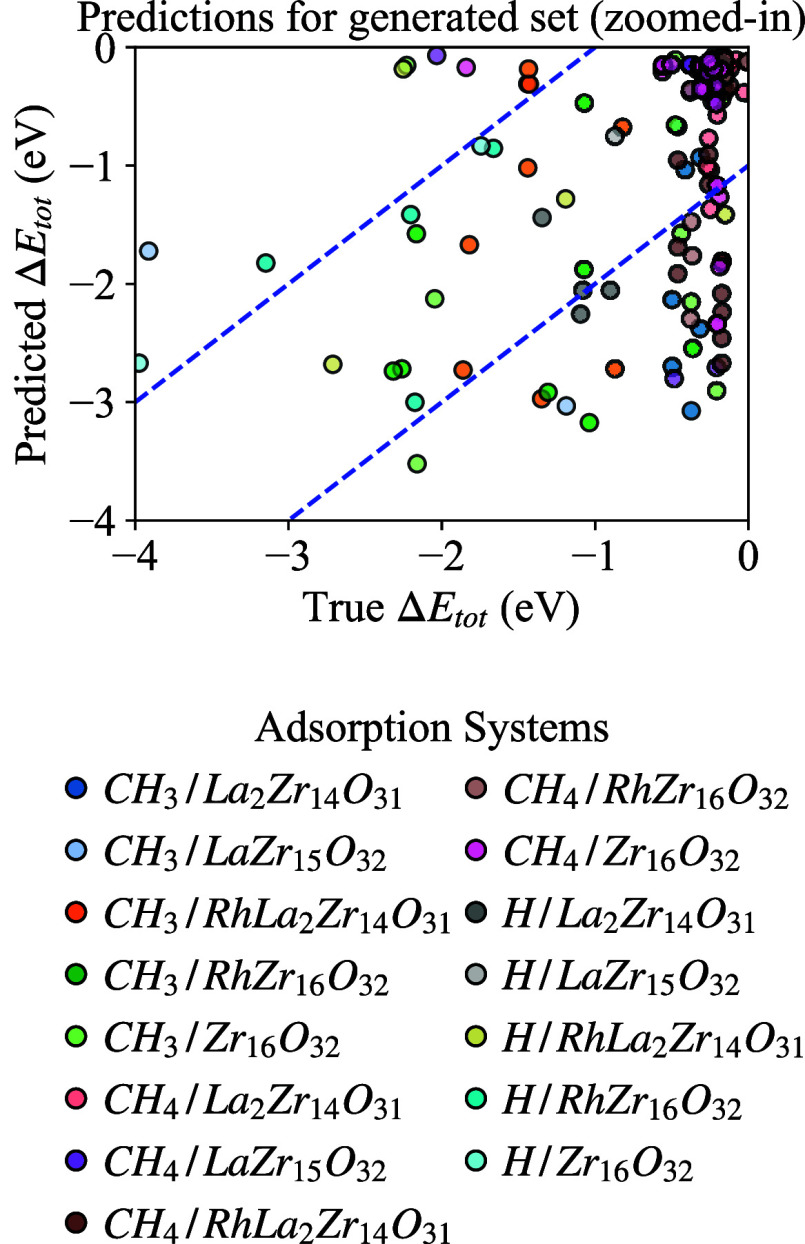
Comparison of the predicted relative energy values from the MLP against the true values from the generated set. The blue diagonal lines represent the 1 eV threshold, which corresponds to the average MAE of the network on this data set. To highlight the majority of the data and reduce the impact of outliers, the plots have been cropped.

**Table 5:** MAE Calculated between the Predictions of the Large MLP Network and the Actual Values from the Generated Adsorption Data Set

system	count	MAE
CH_3_/La_2_Zr_14_O_31_	9	2.090
CH_3_/LaZr_15_O_32_	9	2.103
CH_3_/RhLa_2_Zr_14_O_31_	10	1.148
CH_3_/RhZr_16_O_32_	8	1.268
CH_3_/Zr_16_O_32_	10	1.010
CH_4_/La_2_Zr_14_O_31_	40	0.240
CH_4_/LaZr_15_O_32_	27	0.400
CH_4_/RhLa_2_Zr_14_O_31_	24	0.922
CH_4_/RhZr_16_O_32_	13	0.864
CH_4_/Zr_16_O_32_	35	1.268
H/La_2_Zr_14_O_31_	4	1.220
H/LaZr_15_O_32_	4	2.552
H/RhLa_2_Zr_14_O_31_	4	0.863
H/RhZr_16_O_32_	4	0.937
H/Zr_16_O_32_	6	2.426

The results indicate that the model has difficulty achieving a close to validation performance. This discrepancy is primarily attributed to the previously unseen adsorbate–adsorbent orientations present in the new data sets. It is essential to emphasize that both the training and test sets consisted of real-world data and were unable to cover all of the training space required to generalize for all newly generated data. Despite this limitation, the model remains valuable for performing the preliminary screening of arbitrary systems, offering insights into configurations of interest for further investigation using computationally expensive DFT calculations.

## 5 Conclusions

In this work, we addressed the challenge of using real-world adsorption data sets that were not originally designed for ML. These former data sets present difficulties due to nonstandard formatting, class imbalance, and system heterogeneity. However, we successfully trained an effective predictive model by preprocessing the data and selecting the appropriate ML methods. We first developed Cut-SOAP, a chemical descriptor specifically tailored to adsorption systems. This descriptor rivals the accuracy of naïve SOAP implementations while requiring significantly fewer features, making it well suited for large-scale data sets due to its reduced computational cost. By examining key SOAP hyperparameters and their influence on feature quality, we converted FHI-aims output data into a tabular format compatible with ML models.

Subsequently, we evaluated several combinations of neural network hyperparameters for the MLP. The selected model achieved an average MAE below 0.1 eV in the test set. To further assess its generalization capability, we applied the model to newly generated adsorption systems exhibiting orientation variability, thereby introducing spatial configurations not present in the training data. Even in this more complex scenario, the model achieved a general mean absolute error of less than 1 eV, demonstrating its practical utility as a fast screening method for relative energy interactions. All components of this workflow have been integrated into a user-friendly console interface, allowing specialists to efficiently screen a large number of adsorption systems and identify those with promising relative energy values. In general, this study highlights the potential of combining general-purpose data sets with customized descriptors and ML techniques to accelerate material discovery.

## Supplementary Material



## Data Availability

As mentioned, all DFT calculations were performed using the FHI-aims package, which is available under a nonfree academic license. Additional information about the software can be found at https://fhi-aims.org/. The source code associated with the tools developed in this work is publicly available at https://github.com/CIDAG/Adspt and the training data at https://zenodo.org/records/17238392.

## Footnotes

a
https://singroup.github.io/dscribe/latest/.

## Notes

Data employed for the figures, specifics on self-consistent electronic densities, optimization parameters, and complementary analyses and additional technical details (PDF)

## Nomenclature

**Abbreviations**
AA-SOAP: all-atoms SOAP
ACSF: atom-centered symmetry functions
BEP: Bell–Evans–Polanyi
CM: Coulomb matrix
DFT: density functional theory
EVCM: eigenvalues of the Coulomb matrix
FHI-aims: Fritz–Haber Institute *ab initio* materials simulation
LMBTR: local MBTR
MAE: mean absolute error
MBTR: many-body tensor representation
ML: machine learning
MLP: multilayer perceptron
MS: materials science
PBE: Perdew–Burke–Ernzerhof
PE: potential energy
RMSE: root mean square error
ReLU: rectified linear unit
SGD: stochastic gradiant descent
SMILES: simplified molecular input line entry specification
SOAP: smooth overlap of atomic positions
SP-SOAP: single-point SOAP
TS: Tkatchenko–Scheffler
