# Supplementary material for: Cut-SOAP: A Machine Learning Descriptor for Rapid Screening of Molecular Adsorption Energetics
Source: ACS Omega. 2026 Jan 26;11(5):7948–58. doi: 10.1021/acsomega.5c10055 (PMC12902971; doi:10.1021/acsomega.5c10055)
Supplement: Supplementary file 1 [file ao5c10055_si_001.pdf]

Electronic Supporting Information File: Cut-SOAP: A Machine Learning Descriptor for Rapid Screening of Molecular Adsorption Energetics

Felipe V. Calderan,† Karla F. Andriani,‡ Priscilla Felício-Sousa,¶ Gabriel A. Pinheiro,† Juarez L. F. Da Silva,¶ and Marcos G. Quiles*,†

†*Institute of Science and Technology, Federal University of São Paulo, Av. Cesare Lattes,* 1201, 12247-014*, São José dos Campos, SP, Brazil*

‡*Departament of Exact Sciences, State University of Santa Cruz, Rod. Ilhéus-Itabuna,* 45662-900*, Ilhéus, BA, Brazil*

¶*São Carlos Institute of Chemistry, University of São Paulo, Av. Trabalhador São-Carlense* 400, 13560-970*, São Carlos, SP, Brazil*

E-mail: quiles@unifesp.br

Contents

S-1 Theoretical Approach and Computational Details: Total Energy Calculations S-3

S-2 Dataset Analysis S-4

S-3 Dataset Augmentation S-8

S-4 All Descriptors comparison S-9

S-5 MLP topologies and hyperparameters comparison S-12

S-6 Details for generated systems S-13

References S-14

S-1 THEORETICAL APPROACH AND COMPUTATIONAL DETAILS: TOTAL ENERGY CALCULATIONS

Our *ab initio* total energy calculations were based on spin-polarized Density Functional Theory (DFT) employing the semi-local exchange-correlation energy functional introduced by Perdew–Burke–Ernzerhof (PBE).[1] In order to enhance the depiction of weak long-range van der Waals (vdW) interactions, which are essential for adsorbed systems, especially in the context of physisorption and weak chemisorption phenomena, we employed the vdW formulation proposed by Tkatchenko–Scheffler (TS).[2] This adjustment achieves the DFT-corrected total energy by integrating the attractive vdW energy with the conventional DFT-PBE energy, termed PBE-TS. The Kohn–Sham (KS) equations were addressed using the Fritz–Haber Institute *ab initio* molecular simulations (FHI-aims) package,[3] wherein the all-electron solution for the KS equations was derived employing the scalar-relativistic framework within the zero-order regular approximation.[4] The KS orbitals were expanded into numerical atom-centered orbitals (NAOs), hierarchically constructed from a minimal basis set of free-atom orbitals progressing to the second basis set enhancement, identified as *light-tier2* (according to FHI-aims nomenclature),[5]. However, for adsorbed systems with numerous atoms, the initial set enhancement, i.e., *light-tier1*, was utilized.

Different criteria for the self-consistent electronic density were employed because of the heterogeneity of the systems. However, in most of them the self-consistent electronic density for the adsorbed systems was obtained once the following criteria were reached, that is, the total energy and atomic force criteria were smaller than 2.5×10^{-6} eV and 2.5×10^{-5} eV Å^{-1}, respectively. The modified Broyden-–Fletcher-–Goldfarb-–Shanno (BFGS) algorithm was used to optimize the atomic forces on each atom, and the equilibrium structures were determined when the atomic forces on each atom were smaller than 2.5×10^{-4} eV Å^{-1}. For all total energy calculations, we employed a Gaussian broadening parameter of 10 meV to achieve the correct occupation of electronic states, which is particularly crucial for the cluster due to the small energy separation between

the highest occupied molecular orbital (HOMO) and the lowest unoccupied molecular orbital (LUMO).

S-2 DATASET ANALYSIS

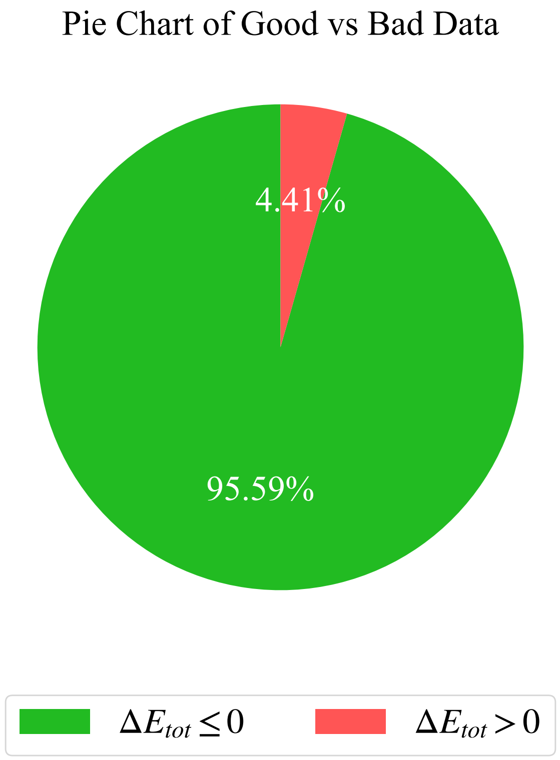

Figure S-1. Visualization of the valid and invalid data amounts.

The data set contains a substantial variety of adsorbates (in our case, a series of molecules), as shown in Figure S-1, as well as adsorbents (metallic and oxide substrates), as represented in Figure S-2.

Table S-1. Count of adsorbate types in the dataset, as determined by the observed adsorbate–adsorbent combinations (amounts to the total number of valid systems — 431638).

Molecule	Count	Molecule	Count	Molecule	Count
CH_4	145,318	CH_2	7444	O_2	329
CH_3	99,080	H_2	7280	C_2H_4O	301
H	32,395	CH	4368	C_2H_6O	274
CH_4O	23,200	C	3100	O	263
CO	22,702	C_2H_2O	2188	CH_4O_2	196
CHO	22,009	C_2H_3O	1292	C_2H_5O	137
CH_3O	18,715	HO	795	H_2O	130
CHO_2	17,805	C_2H_2O	661	H_2O_2	58
CH_2O	12,637	N	468	S	30
CO_2	8109	O_2S	338	H_3N	16

Table S-2. Count of adsorbent types in the dataset, as determined by the observed adsorbate–adsorbent combinations (amounts to the total number of valid systems — 431638).

Subs.	Count	Subs.	Count	Subs.	Count	Subs.	Count
Fe13	52,769	Co15	5930	Ni10	4107	Co6	1972
Cu13	41,164	Cu8	5693	Co11	4077	Ni7	1934
Co13	38,047	Ni15	5672	Fe11	4031	Co7	1910
Ni13	34,840	Fe14	5335	Co10	4006	Ni6	1701
La2O31Zr14	17,239	Co14	5259	Ni11	3637	Fe5	1510
O32Zr16	16,475	Cu11	5137	Co8	3484	Co5	1454
LaO32Zr15	13,176	Ni14	5038	Fe9	3364	Ni5	1198
Ga8	12,600	Fe8	5008	Ni9	3257	Co4	1048
Ni8	12,393	Cu12	4913	Fe10	3219	Au8	892
Ga3Ni5	10,279	Ni12	4878	Cu7	3143	Cu4	854
La2O31RhZr14	9336	Cu55	4748	Co9	3126	Ni4	598
O32RhZr16	8876	Co12	4649	Cu6	3031	Fe4	575
Cu15	8810	Cu9	4608	Fe7	2904	Cu42Zn13	469
Fe15	7216	Fe12	4400	Cu5	2139	Pd8	239
Cu14	6570	Cu10	4369	Fe6	2135	Ru8	197

Table S-3. Count of distinct references for each adsorbate type in the dataset. Each reference corresponds to a known geometric configuration of the corresponding molecule.

Molecule	Count	Molecule	Count	Molecule	Count
CH3	81	CH2	9	C2H5O	4
CH4	80	CHO2	9	H2O2	1
H	73	CH	9	H2O	1
C2H3O	20	C	9	H3N	1
CO	20	CH4O	9	O2S	1
CHO	18	H2	8	S	1
CH2O	12	C2H4O	8	N	1
CH3O	12	HO	5	O2	1
C2H2O	12	O	5	C2H6O	1
CO2	11	C2HO	4	CH4O2	1

Table S-4. Count of distinct references for each adsorbent type in the dataset. Each reference corresponds to a known geometric configuration of the corresponding substrate.

Subs.	Count	Subs.	Count	Subs.	Count	Subs.	Count
O32Zr16	33	Cu8	4	Ni7	3	Cu12	3
Fe13	29	Co8	4	Fe11	3	Cu14	3
Ni13	29	Co9	3	Fe7	3	Ni4	3
Co13	29	Co4	3	Fe10	3	Cu6	3
Cu13	29	Co10	3	Fe9	3	Cu7	3
Cu42Zn13	28	Co14	3	Fe6	3	Ni10	3
Cu55	28	Co5	3	Ni12	3	Ni11	3
La2O31Zr14	24	Co7	3	Fe5	3	Cu9	3
La2O31RhZr14	12	Co12	3	Ni5	3	Cu5	3
O32RhZr16	12	Co15	3	Ni6	3	Cu11	3
Ni8	12	Fe14	3	Ni15	3	Cu15	3
LaO32Zr15	12	Co6	3	Ni14	3	Fe4	2
Ga3Ni5	8	Co11	3	Fe12	3	Ru8	1
Ga8	8	Cu4	3	Fe15	3	Pd8	1
Fe8	4	Ni9	3	Cu10	3	Au8	1

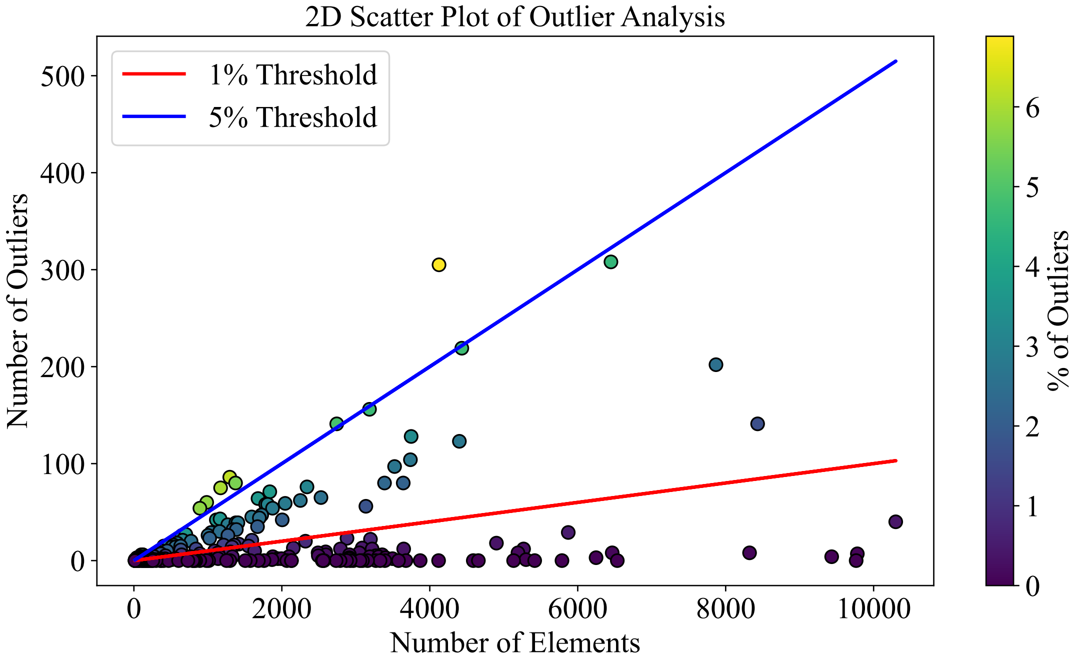

Figure S-2. This chart represents the ratio between number of elements and outliers in respect to ΔE_{tot} for each type of system. The lines represent thresholds of 1 % and 5 %.

During our experiments, we removed elements with positive relative energy interactions (ΔE_{tot}) from the dataset due to their unwanted physicochemical implications (as the distance between atoms goes to infinity, the energy approaches zero). Of the 451,535 data points, 431,638 agree with the imposed criterion. This represents a loss of less than 5 % of the initial data, which is not largely significant (see Figure S-1), due to the large amount of data. Further plots and analyses will only consider the valid data.

As our dataset is originally composed of real data that was employed for alternative purposes and not specifically curated for Machine Learning applications, it exhibits significant imbalances concerning the distribution of each type of system (combinations of molecules and substrates). Consequently, it is imperative to examine the impact of these imbalances on various components of the methodology. Figure S-2 illustrates, for each type of system, the correlation between the number of elements belonging to that type and the number of outliers with respect to their ΔE_{tot}. Outliers are identified as entries with a Z-Score exceeding 3. The lower blue line denotes the 1 % threshold, whereas the upper line indicates 5 %. With the exception of CO Ni_3 (represented by

the yellow dot at $X \approx 4000$ and $Y \approx 300$), the prevalence of outliers is generally within acceptable limits.

S-3 DATASET AUGMENTATION

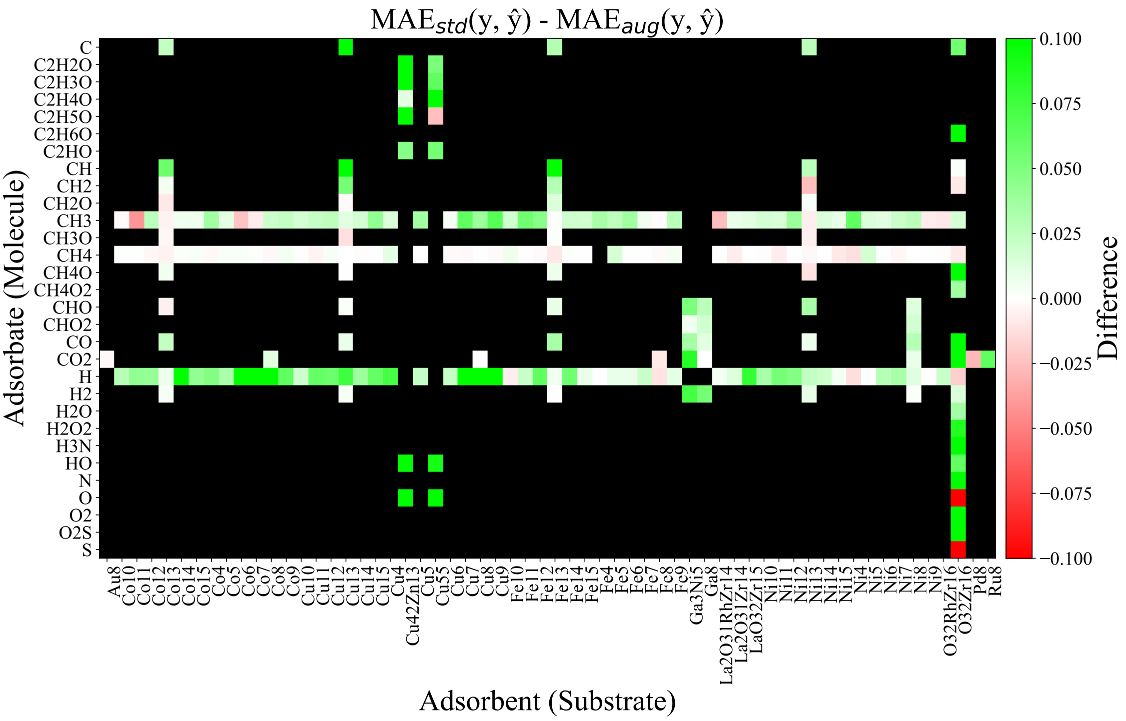

Figure S-3. This figure displays the overall improvement in prediction quality post-oversampling augmentation.

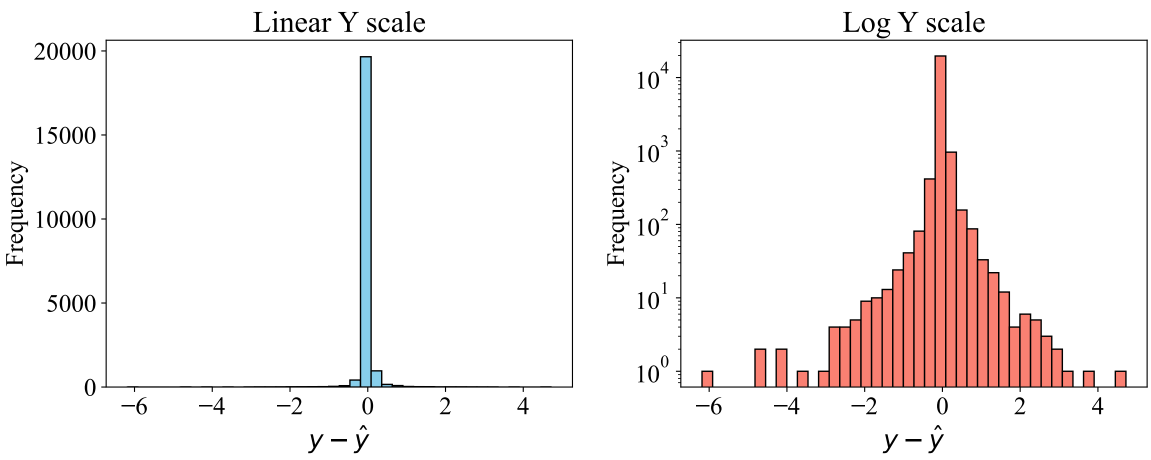

Figure S-4. This figure shows the error distribution for predictions made using Cut-SOAP 2 and an MLP network. The left chart uses a linear Y-axis scale, while the right uses a logarithmic one.

Continuing the discussion on imbalances, in an effort to enhance the results of the machine learning models, random oversampling was applied to the underrepresented classes within the data set. This augmentation technique ensures that each combination of molecule and substrate accounts for at least 1 % of the overall data set. Employing the Cut-Soap 2 descriptor (Table S-5) and the Standard MLP architecture (Table S-6), we evaluated the impact of augmentation on prediction quality. As illustrated in Figure S-3, the augmentation method has been observed to yield predominantly positive effects on the data set. Furthermore, Figure S-4 depicts the distribution of error $\hat{y} - y$, with the majority of errors clustered in close proximity to 0. The model demonstrated a capacity for producing accurate predictions, with significant errors occurring infrequently. Instances of larger errors typically involved underrepresented systems, where the network was less effective in learning the underlying patterns compared to more adequately represented combinations.

S-4 ALL DESCRIPTORS COMPARISON

Table S-5 is a reference for all hyperparameters used for the tests executed, for future reproducibility reasons. These parameters are set in JSON files that are used by the software to determine how the descriptors should initialize and behave.

Table S-5. JSON settings for each descriptor in the comparison.

**AA-Soap**	**Cut-Soap 0**	**Cut-Soap 2**
{<br>"site_size": 6,<br>"r_cut": 3.5,<br>"n_max": 2,<br>"l_max": 1,<br>"sigma": 0.5,<br>"periodic": "false"<br>}	{<br>"site_size": 6,<br>"r_cut": 2.5,<br>"n_max": 1,<br>"l_max": 0,<br>"sigma": 0.5,<br>"periodic": "false"<br>}	{<br>"site_size": 6,<br>"r_cut": 3.5,<br>"n_max": 2,<br>"l_max": 1,<br>"sigma": 0.5,<br>"periodic": "false"<br>}
**Soap**	**SP-Soap**	**Cut-EVCM**
{<br>"r_cut": 3.5,<br>"n_max": 2,<br>"l_max": 1,<br>"sigma": 0.5,<br>"periodic": "false"<br>}	{<br>"r_cut": 10,<br>"n_max": 2,<br>"l_max": 1,<br>"sigma": 0.5,<br>"periodic": "false"<br>}	{<br>"site_size": 6<br>}

Naturally, the more detailed a descriptor is, the higher the computational costs typically are to build and process it. There are many factors that contribute to how detailed a descriptor is able to capture the characteristics of a chemical system. One of them is the number of generated features, although it is of utmost importance to perform a cost-benefit analysis. Generally, a very large number of features tends to have diminishing returns and can even be detrimental to the final prediction quality, due to model overfitting, therefore a more compact model with higher-quality features is highly preferred. Figure S-5 shows how the build time escalates with the number of features of different descriptors.

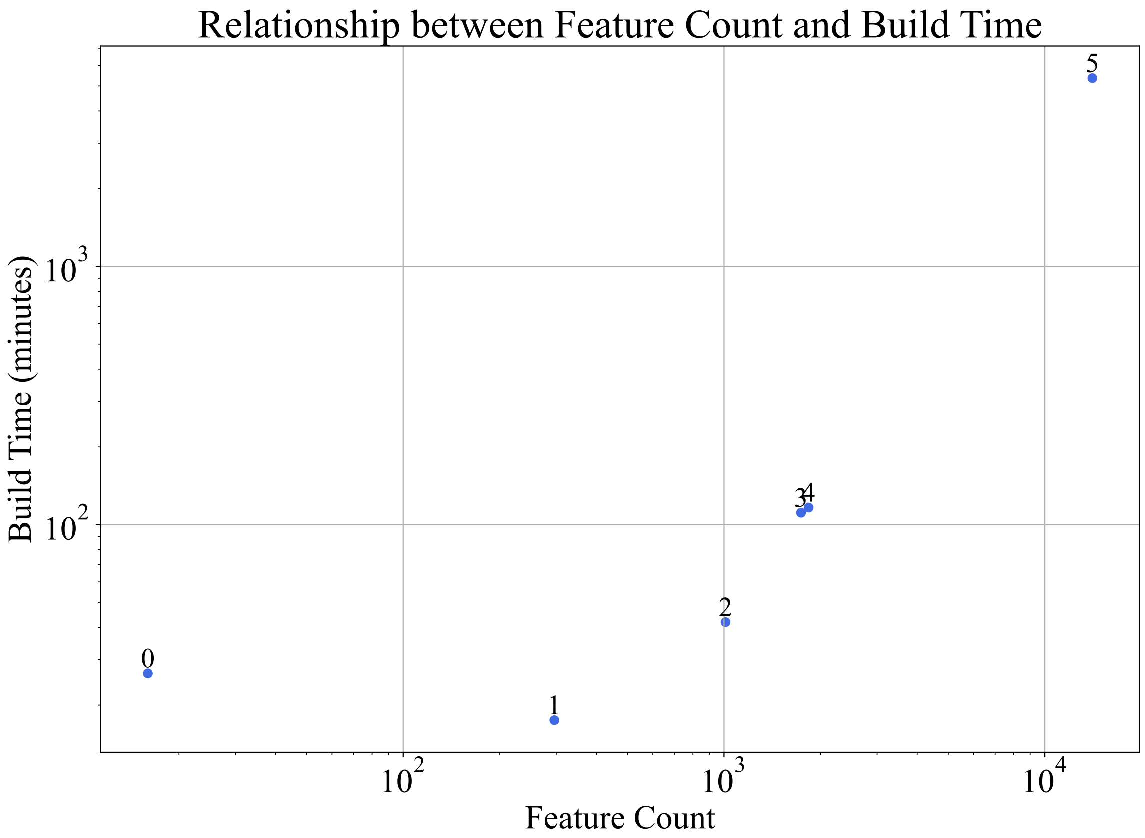

Figure S-5. This figure takes into consideration the following descriptors: (0) Cut-EVCM, (1) Cut-SOAP 0, (2) SP-SOAP, (3) SOAP, (4) Cut-SOAP 2, and (5) AA-SOAP, in respect to the relationship between their build time and number of features produced. Both axes are in log scale.

S-5 MLP TOPOLOGIES AND HYPERPARAMETERS COMPARISON

Table S-6. JSON settings for each MLP in the comparison.

**Standard**	**Flat**
{ "network": {<br>"hidden_layers": [1024, 256, 64, 8],<br>"activation_fn": "ReLU"<br>},<br>"optimization": {<br>"optimizer": "Adam",<br>"optim_params": { "lr": 0.001 }<br>},<br>"training": {<br>"train_size": 0.9,<br>"epochs": 500,<br>"batch_size": 10000<br>} }	{ "network": {<br>"hidden_layers": [64, 8],<br>"activation_fn": "ReLU"<br>},<br>"optimization": {<br>"optimizer": "Adam",<br>"optim_params": { "lr": 0.001 }<br>},<br>"training": {<br>"train_size": 0.9,<br>"epochs": 500,<br>"batch_size": 10000<br>} }
**MiniBatch**	**Large**
{ "network": {<br>"hidden_layers": [1024, 256, 64, 8],<br>"activation_fn": "ReLU"<br>},<br>"optimization": {<br>"optimizer": "Adam",<br>"optim_params": { "lr": 0.001 }<br>},<br>"training": {<br>"train_size": 0.9,<br>"epochs": 500,<br>"batch_size": 64<br>} }	{ "network": {<br>"hidden_layers": [1536, 1024, 512, 256, 64, 8],<br>"activation_fn": "ReLU"<br>},<br>"optimization": {<br>"optimizer": "Adam",<br>"optim_params": { "lr": 0.001 }<br>},<br>"training": {<br>"train_size": 0.9,<br>"epochs": 500,<br>"batch_size": 10000<br>} }
**Patient**	**Combo**
{ "network": {<br>"hidden_layers": [1024, 256, 64, 8],<br>"activation_fn": "ReLU"<br>},<br>"optimization": {<br>"optimizer": "Adam",<br>"optim_params": { "lr": 0.0001 }<br>},<br>"training": {<br>"train_size": 0.9,<br>"epochs": 5000,<br>"batch_size": 10000,<br>"patience": 50<br>} }	{ "network": {<br>"hidden_layers": [1536, 1024, 512, 256, 64, 8],<br>"activation_fn": "ReLU"<br>},<br>"optimization": {<br>"optimizer": "Adam",<br>"optim_params": { "lr": 0.0001 }<br>},<br>"training": {<br>"train_size": 0.9,<br>"epochs": 5000,<br>"batch_size": 64,<br>"patience": 50<br>} }

S-6 DETAILS FOR GENERATED SYSTEMS

Table S-7 provides additional information about the generated adsorption dataset. The simplified table in the main body of this work does not account for elements with positive ΔE_{tot}, since the model was explicitly designed without training on systems with this characteristic. This is clear from the reduction in MAE when only elements with negative ΔE_{tot} are considered, particularly in systems with a higher Pos/Neg ratio.

Table S-7. Complete table for the predictions of the large MLP network and the actual values from the generated adsorption dataset. **"Pos"** refers to entries with positive relative energy, while **"Neg"** is the opposite. **"MAE (Neg)"** only considers entries with negative relative energy, **"Train"** refers to the number of entries in the training set that matches the specified system. **"Train (aug)"** is the same, but after oversampling. Finally, **"Test"** is the number of entries in the original test set.

System	Pos	Neg	Total	MAE	MAE (Neg)	Train	Train (aug)	Test
$CH_3/La_2Zr_{14}O_{31}$	0	9	9	2.090	2.090	4658	4658	243
$CH_3/LaZr_{15}O_{32}$	0	9	9	2.103	2.103	5501	5501	286
$CH_3/RhLa_2Zr_{14}O_{31}$	0	10	10	1.148	1.148	2895	4100	153
$CH_3/RhZr_{16}O_{32}$	2	8	10	1.268	1.100	2601	4100	133
$CH_3/Zr_{16}O_{32}$	0	10	10	1.010	1.010	3327	4100	169
$CH_4/La_2Zr_{14}O_{31}$	1	40	41	0.240	0.154	9308	9308	472
$CH_4/LaZr_{15}O_{32}$	0	27	27	0.400	0.400	3913	4100	206
$CH_4/RhLa_2Zr_{14}O_{31}$	1	24	25	0.922	0.944	2935	4100	155
$CH_4/RhZr_{16}O_{32}$	9	13	22	0.864	0.403	2903	4100	153
$CH_4/Zr_{16}O_{32}$	1	35	36	1.268	1.038	6125	6125	324
$H/La_2Zr_{14}O_{31}$	1	4	5	1.220	0.845	2429	4100	129
$H/LaZr_{15}O_{32}$	0	4	4	2.552	2.552	3106	4100	164
$H/RhLa_2Zr_{14}O_{31}$	0	4	4	0.863	0.863	3038	4100	160
$H/RhZr_{16}O_{32}$	0	4	4	0.937	0.937	2932	4100	154
$H/Zr_{16}O_{32}$	0	6	6	2.426	2.426	2929	8200	154

Figure S-6 presents the predicted versus true ΔE_{tot} values for all data points in the generated set. Aside from a few outliers, such as two $CH_4/Zr_{16}O_{32}$ systems and a handful of others, the majority of predictions lie within the 1 eV error margins. While this error is higher than the average MAE observed on the test set, the result is expected. Unlike the test set, which consists of a random split from the training data and thus shares similar orientations, the generated set includes systems with diverse molecular-substrate orientations not seen during training.

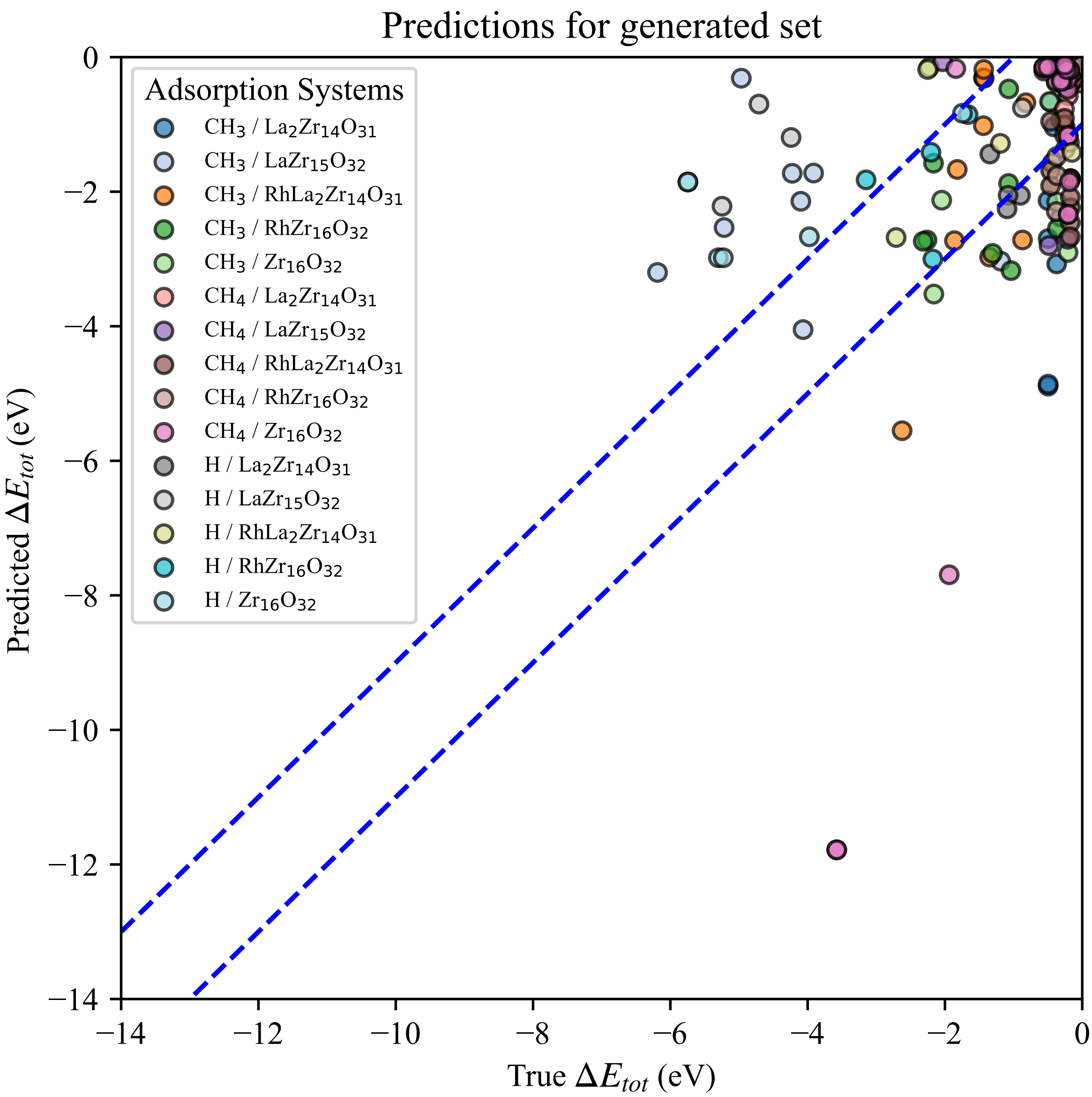

Figure S-6. This chart presents the full version of Figure 4 from the main text, including all data points without cropping.

References

1 Perdew, J. P.; Burke, K.; Ernzerhof, M. Generalized Gradient Approximation Made Simple. *Phys. Rev. Lett.* **1996**, *77*, 3865–3868, DOI: 10.1103/physrevlett.77.3865.

2 Tkatchenko, A.; Scheffler, M. Accurate Molecular van Der Waals Interactions From Ground-state Electron Density and Free-atom Reference Data. *Phys. Rev. Lett.* **2009**, *102*, 073005, DOI: 10.1103/physrevlett.102.073005.

3 Blum, V.; Gehrke, R.; Hanke, F.; Havu, P.; Havu, V.; Ren, X.; Reuter, K.; Scheffler, M. *Ab initio* Molecular Simulations With Numeric Atom-centered Orbitals. *Comput. Phys. Commun.* **2009**, *180*, 2175–2196, DOI: `10.1016/j.cpc.2009.06.022`.

4 van Lenthe, E.; Snijders, J. G.; Baerends, E. J. The Zero-Order Regular Approximation for Relativistic Effects: The Effect of Spin–Orbit Coupling in Closed Shell Molecules. *J. of Chem. Phys.* **1996**, *105*, 6505–6516, DOI: `10.1063/1.472460`.

5 Havu, V.; Blum, V.; Havu, P.; Scheffler, M. Efficient Integration for All-electron Electronic Structure Calculation Using Numeric Basis Functions. *J. Comput. Phys.* **2009**, *228*, 8367–8379, DOI: `10.1016/j.jcp.2009.08.008`.
